# Temporal Beta Diversity of Bacteria in Streams: Network Position Matters But Differently for Bacterioplankton and Biofilm Communities

**DOI:** 10.1007/s00248-025-02522-3

**Published:** 2025-04-12

**Authors:** Kaisa-Leena Huttunen, Jacqueline Malazarte, Jussi Jyväsjärvi, Kaisa Lehosmaa, Timo Muotka

**Affiliations:** 1 Finnish Environment Institute, Nature Solutions Unit, Oulu, Finland; 2 University of Oulu, Ecology and Genetics Research Unit, Oulu, Finland; 3 University of Oulu, Water, Energy, and Environmental Engineering Research Unit, Oulu, Finland; 4 Centre for Economic Development, Transport and the Environment of North Ostrobothnia, Oulu, Finland; 5 University of Oulu, Oulanka Research Station, Kuusamo, Finland

**Keywords:** Aquatic bacteria, Community composition, Headwaters, Stream network, Temporal variability, Stream-forest linkages

## Abstract

Concern about biodiversity loss has yielded a surge of studies on temporal change in α-diversity, whereas temporal β-diversity has gained less interest. We sampled bacterioplankton, biofilm, and riparian soil bacteria repeatedly across the open-water season in a pristine stream network to determine the level of temporal β-diversity in relation to stream network position and environmental variability. We tested the hypothesis that aquatic bacterial communities in isolated and environmentally heterogenous headwaters exhibit high temporal β-diversity while the better-connected and environmentally more stable mainstem sections support more stable communities, and soil communities bear no relationship to network position. As expected, temporal β-diversity decreased from headwaters toward mainstems for bacterioplankton. Against expectations, an opposite pattern was observed for biofilm. For bacterioplankton, temporal β-diversity was positively related to temporal variability in water chemistry. For biofilm bacteria, temporal variability was negatively related to variability in temperature. Temporal β-diversity of soil communities did not show any response to stream network position, but was strongly related to variability in the soil environment. The two aquatic habitats and riparian soils supported distinctly different bacterial communities. The number of ASVs shared between the soil and the aquatic communities decreased along the network, and more so for bacterioplankton. The higher temporal variability of bacterial communities in the headwaters likely results from temporally variable input of propagules from riparian soil, emphasizing the role of land–water connection and network position to bacterioplankton community composition. Overall, bacterial communities exhibited high temporal variability, highlighting the importance of temporal replication to fully capture their network-scale biodiversity.

**Supplementary Information:**

The online version contains supplementary material available at 10.1007/s00248-025-02522-3.

## Introduction

Variation of biological communities across sites or along environmental gradients, i.e., spatial β-diversity, has been a fundamental concept in community ecology since the seminal works by Whittaker in 1960 [1, 2]. Much of the research on spatial β-diversity uses snapshot data on community composition at a collection of sites, although it is generally recognized that metacommunities are highly dynamic and therefore only studies that analyze β-diversity over time capture the full range of variation in community composition [3, 4]. The concern about biodiversity loss has generated a surge of studies on temporal change in α-diversity whereas temporal β-diversity, change in community composition in repeated surveys through time [5], has gained broad interest only recently. Several recent studies have shown local species richness to be rather invariant through time while compositional changes may be substantial [6, 7]. However, these studies have used compilations of long-term data sets across several ecosystems and are therefore biased towards higher taxonomic groups for which such data are available.

Rivers occur as hierarchically branching networks where the abiotic environment varies predictably along the upstream–downstream continuum, and different network positions support biological assemblages driven by largely distinct assembly mechanisms [8–11]. It is commonly assumed that the upmost headwaters receive little immigration and are environmentally heterogenous, exhibiting high among-site community variability (high spatial β-diversity). In contrast, the better-connected and environmentally more stable mainstem sections receive great numbers of immigrants from both upstream and downstream directions, thus supporting less variable communities (lower β-diversity) [12–14]. However, studies testing these hypotheses are taxonomically biased, focusing on diatoms [15, 16], benthic invertebrates [8, 9], or fish [10, 15]. These groups disperse either mainly or exclusively along stream corridors, and in the case of invertabrates and fish, both upstream and downstream direction. However, patterns (and direction) of dispersal may be vastly different for microbial organisms, in which case expectations about patterns of β-diversity along the river network may also differ.

A number of studies have shown that microbial α-diversity in stream networks decreases from the upmost headwaters toward downstream sections, particularly for bacterioplankton [17–19] but also, although less consistently so, for benthic biofilm bacteria [20, 21]. Whether network position also controls bacterial β-diversity remains less explored, and practically no previous studies have addressed temporal β-diversity in relation to network position. Spatial studies have suggested, however, that microbial β-diversity is highest among headwater sites [19, 20, 22] and decreases with dendritic distance from upstream sources towards downstream direction in both bacterioplankton and biofilm communities [11].

Headwater bacterioplankton assemblages are unlikely to be dispersal-limited, because they receive massive amounts of propagules from riparian soils [17, 18, 23], particularly during rainfall-induced floods [19, 24]. Unpredictable variation in the timing and extent of rainfall and scouring flows might be expected to induce temporal variability in headwater bacterioplankton while no such mechanism should be expected for biofilm communities that are consistently under strong environmental selection, favoring taxa that possess traits beneficial in benthic habitats [25, 26]. Indeed, many studies have shown that biofilm community composition is largely independent of the taxa pool present in the overlying water column [11, 21, 26]. Consequently, biofilm communities could be expected to exhibit lower temporal β-diversity than bacterioplankton, irrespective of network position.

We used data on the distribution and relative abundance of bacterial taxa in 13 sites, sampled seven times each from early July to October 2018, in a subarctic stream network to explore hypotheses about temporal variability in bacterioplankton, biofilm, and riparian soil communities. Network-scale variation in spatial patterns of α-richness and community composition were addressed in an earlier paper by Malazarte et al. [21] and we therefore focus here on temporal aspects of community variability, particularly the effects of network position on temporal β-diversity. We hypothesized that (i) temporal β-diversity of bacterioplankton peaks in headwater sites where the stream environment, particularly water chemistry, also exhibits the strongest temporal variability; temporal β-diversity should then decrease with increasing distance from the stream source. Next, we expected (ii) the benthic habitat to select similar taxa in all network positions; consequently, biofilm communities should vary less through time than does bacterioplankton, with little difference in temporal β-diversity among network positions. As riparian soil communities should not bear any relationship to network position, we expected (iii) temporal variability of soil communities to be controlled only by local edaphic factors, particularly soil pH [27, 28] and nutrient concentrations [29]. Finally, we expected (iv) the similarity of bacterioplankton to soil communities to be highest overall, and vary most through time, in headwaters reflecting their close water-land linkage, whereas (v) benthic biofilm was expected to show a generally weaker (if any) relationship with soil communities.

## Methods

### Study Stream and Sampling Protocol

Our study focused on River Riisijoki, a near-pristine river network with a catchment area of 27.6 km^2^, mostly located within the Riisitunturi National Park in northeastern Finland. The river runs through coniferous-dominated forests and peatlands before draining into Lake Kitkajärvi. The river is ice-covered from early November to early May. Mean annual discharge is 0.34 m^3^ s^−1^, with a peak of ca. 5 m^3^ s^−1^ in late May. Water is slightly acidic (pH 5.4–6.7), oligo-to-mesotrophic (tot-P 8–25 μg L^−1^), and mesohumic (DOC 8–17 mg L^−1^) (for a more detailed description of the study system, see [21, 30]).

**Fig. 1:**
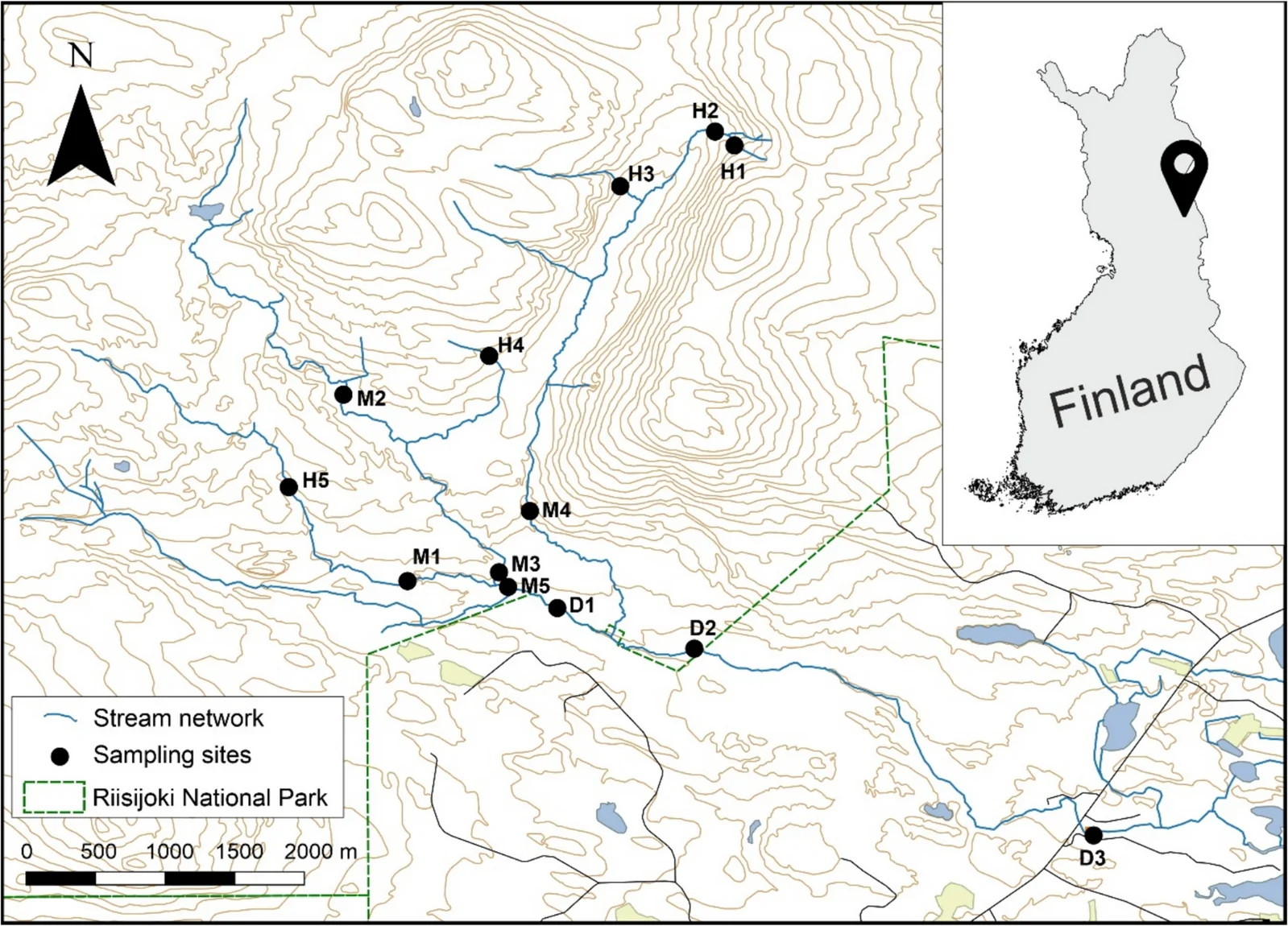
Location of the study sites in the Riisijoki river network in northeastern Finland. H = headwater (1 st order), M = mid-stream (2nd order), and D = downstream (3rd order) sites. The map was first published in Malazarte et al. [21]

We sampled 13 sites from the upmost headwaters to the downmost site close to the lake inlet (Fig. 1). Headwater sites are first-order streams (*n* = 5; stream width 0.5–1.5 m), mid-stream sites second-order streams (*n* = 5; 2–3 m), and downstream sites third-order streams (*n* = 3; 5–8 m) based on Strahler stream order. For each study site, we calculated dendritic distance along the stream channel from the upstream source to describe its position in the river network. The maximum river length (i.e., dendritic distance from upstream source to the downmost site) is ca. 10 km. Sampling was carried out in about 2-week intervals from early July to October 2018 with overall seven sampling occasions. Local daily mean temperature during the sampling period ranged from − 5 to 25 °C and daily precipitation sum from 0 to 45 mm (Fig. S1).

Benthic biofilm was sampled from a riffle section at each site as a pooled sample from the upper side of four randomly selected cobble-sized stones by wiping them carefully with sterile celluse sponges (Speci-Sponge®, Whirl–Pak, Nasco) allowing convenient and sterile sampling for biofilm bacteria in the field [31]. All four stones per site were wiped with one sponge, which was then placed in a sterile 50-mL centrifuge tube. For bacterioplankton, three replicate samples were collected from the water column into sterile 50-mL centrifuge tubes. In addition, to allow a direct comparison of bacterioplankton and biofilm communities with riparian soil communities, we collected soil samples at each site on each sampling occasion. Soil samples were collected at a 1-m distance from the stream edge to a depth of 10 cm using a 2.5-cm diameter soil corer. Four replicate samples were pooled to obtain a composite sample for each site. All bacterial samples were immediately placed on dry ice and stored to − 20 °C within a few hours until further processing.

Concurrently with bacterial sampling, we collected water samples at each site into 500-mL plastic bottles and analyzed those for pH, total phosphorus (TotP μg L^−1^), nitrate-nitrite nitrogen (NO_3_-NO_2_-N μg L^−1^), and specific ultraviolet absorbance (SUVA). Samples for TotP and NO_3_-NO_2_-N were filtered (Whatman GF/C) and frozen before colometric analysis with AutoAnalyzer 500 (Seal Analytical). SUVA was used as a surrogate for stream water DOM (dissolved orgaic matter) aromaticity, with higher SUVA values indicating higher content of aromatic carbon [32]. It was calculated by measuring stream water absorbance at the 254 nm (Shimadzu UV- 160 A spectrophotometer) from filtered (Whatman GF/C) subsamples and dividing the values with dissolved organic carbon (DOC) concentration (DOC mg L^−1^), which was measured from filtered and preserved (2 M HCl) water samples by infrared spectrometry with Shimadzu TOC-L CSN analyzer (Shimadzu Scientific Instruments). Soil samples were measured for pH and conductivity from the supernatant after soaking 20-mL soil samples in 50 mL of milli-Q water and for total carbon and nitrogen content (%w/w) from dried and powdered samples (7–8 mg) using FlashSmart CHNS/O Elemental Analyzer (Thermo Fisher Scientific, Bremen, Germany) [33].

Data loggers (WT-HR 1000 mm, TruTrack Ltd., New Zealand) were installed at each site and set to record stream water temperature and water level at 30-min intervals. In data analyses, we used mean values across the 7-day period preceding each biological sample.

### Molecular Analyses and Bioinformatics

DNA was extracted from water (bacterioplankton), sponges (biofilm), and soil. For biofilms, 100 mL of molecular-grade water was added to the sponges and bacteria extracted using laboratory blender (Stomacher® 400 Circulator) at 260 rpm for 2 min. Processed biofilm samples and bacterioplankton (150 mL with three replicates combined) were filtered using sterilized 0.2-μm nylon membrane filters (Whatman, UK) which were cut into pieces before DNA extraction, whereas soil samples (2.5 g) were freeze-dried and grinded in sterilized mortars. DNA for all three sample types were extracted with Qiagen’s DNeasy® PowerSoil® DNA Isolation kit. The 16S rRNA gene was amplified using universal forward 519 F (5′-CAGCMGCCGCGGTAATWC − 3′) and R926 (5′-CCGTCAATTCCTTTRAGTTT- 3′) reverse primers [34]. Triplicate 20-μL polymerase chain reactions (PCRs) contained 10 ng of template DNA, 1 × Phusion GC buffer, 0.5 μM of forward and reverse primers, 0.2 mM dNTP’s, and 0.4 U of Phusion high-fidelity DNA polymerase (S7 Fusion Polymerase, MOBIDIAG, Helsinki, Finland). The amplification conditions were an initial denaturation step at 98 °C for 30 s, followed by 30 cycles of denaturation at 98 °C for 10 s, primer annealing at 64 °C for 30 s, extension at 72 °C for 20 s, and a final extension step at 72 °C for 5 min. The PCR amplicon triplicates were pooled (sample volume after pooling = 60 μl), and the pooled samples purified with AmpureXP PCR purification reagent (Agencourt Bioscience) and quantified using Bioanalyzer DNA- 1000 chips (Agilent Technologies, Palo Alto, CA, USA). Then, each sample was quantified with the PicoGreen dsDNA assay kit (ThermoFisher, Carlsbad, CA, USA) and, based on the quantity, diluted to an appropriate concentration prior to sequencing. Ion Torrent sequencing was applied using Ion Torrent Hi-Q OT2 kit, Ion Torrent Hi-Q View Sequencing kit and 316 v2 chip (ThermoFisher, Carlsbad, CA, USA).

Sequences were processed using QIIME2 (v.2020–8) pipeline [35]. Short reads (< 100 bp) were removed and the remaining reads were demultiplexed with sample-specific barcodes using q2-cutadapt plugin [36]. Single-end demultiplexed, adapter trimmed sequences (range 3754–99,510) were further processed with DADA2 using denoise-pyro option with truncate length of 220 nucleotides [37]. After denoising, four separate Ion Torrent sequencing runs were combined. Amplicon sequence variants (ASVs) were aligned to SILVA 16S version 138.1 Gene Database [38] with primer trimmed data (mean number of denoised reads 8778, range 2244–68,895) using pre-trained classify-sklearn naïve Bayes taxonomy classifier (via q2-feature-classifier [39]). Pretraining of the classifier was done using reference reads first modified to QIIME2 format and then extracted using the primers used in the amplification. Prior to further analyses, singletons, mitochondria, chloroplasts, and unassigned sequences were removed. The data are deposited under BioProject accession number PRJNA821862 in the NCBI BioProject database (https://www.ncbi.nlm.nih.gov/bioproject/).

### Data Analyses

All statistical analyses were done using R version 4.3.0 [40]. Prior to analyses the bacterial data were rarefied to 3352 sequences per sample based on the lower 5 th percentile of sample-specific sequence counts. The samples with less than 3352 sequences (*n* = 14) were included to data analyses in their original form (minimum 2239 sequences). Based on the rarefaction curves “species” gain had plateaued at this sequence depth for most of the samples (Fig. S2). Earlier Lundin et al. [41] showed that 1000 denoised sequences from original 15,000–20,000 sequences per sample were enough to explain 90% the trends in β-diversity. Our final rarefied data set had a total of 14846 ASVs, of which 6471 occurred in bacterioplankton, 6473 in biofilm, and 5142 in soil samples.

Our analyses focused on variation in community composition (Bray–Curtis dissimilarity on relative abundance data) across time, i.e., temporal β-diversity. To quantify site-specific temporal β-diversity for each community type (bacterioplankton, biofilm, soil), we calculated the mean dissimilarity across consecutive sampling times using ASV data (R package “vegan” v.2.2.− 0 [42]). In addition, we used temporal distance decay measured as the slope of linear regression between pairwise dissimilarity values and temporal distance between samples to describe the level of dissimilarity in relation to distance in time separately for each site. To study whether these two measures of temporal community variability were related to network position, we used simple linear regression between temporal turnover or temporal distance decay and distance of a site from the upstream source measured as the dendritic distance along the stream channel. Similarly, linear regressions were used to study whether temporal environmental variability was related to network position and to temporal β-diversity of bacterial communities. For this purpose, we calculated site-specific means for temporal variability in environmental conditions across consecutive samples based on pairwise Euclidian distance on standardized values of pH, TotP, NO_2_ + NO_3_, and SUVA (i.e., water chemistry); water temperature and water level (physical environment); and soil pH, conductivity, TotC and N (soil environment). Partial regressions (linear regression model (using lm in R) for residuals) were used to study the effect of environmental variability (e.g., Euclidian distance for water quality or physical environment) on community variability while controlling for stream network position.

Non-metric multidimensional scaling (NMDS) on Bray–Curtis dissimilarity was used to visualize differences in community composition between biofilm, bacterioplankton and riparian soil communities. Differences among community types were tested using permutational multivariate analysis of variance (PERMANOVA; 999 permutations) [42]. In addition, to explore the relationship between the soil and the two aquatic communities at different network positions, we used three different measures: (i) mean similarity between site/time-specific samples of soil vs. bacterioplankton/biofilm communities using Bray–Curtis distance; (ii) distance between group centroids in the NMDS ordination space with R package “usedist” [43]; and (iii) temporal variation (standard deviation) in community similarity across consecutive sampling times.

To more directly assess the contribution of soil-derived taxa to aquatic communities, we regressed, separately for bacterioplankton and biofilm, the number of taxa shared with soil communities against dendritic distance from the source. For a comparison also the number of taxa shared between biofilm and bacterioplankton was included. Shared ASVs were determined separately for each biofilm and bacterioplankton sample based on ASV occurrences at each site at any sampling time. Finally, we determined the taxonomic identity and relative abundance of the most important bacterial ASVs that were shared between the soil and the each of the two aquatic community types.

## Results

**Fig. 2:**
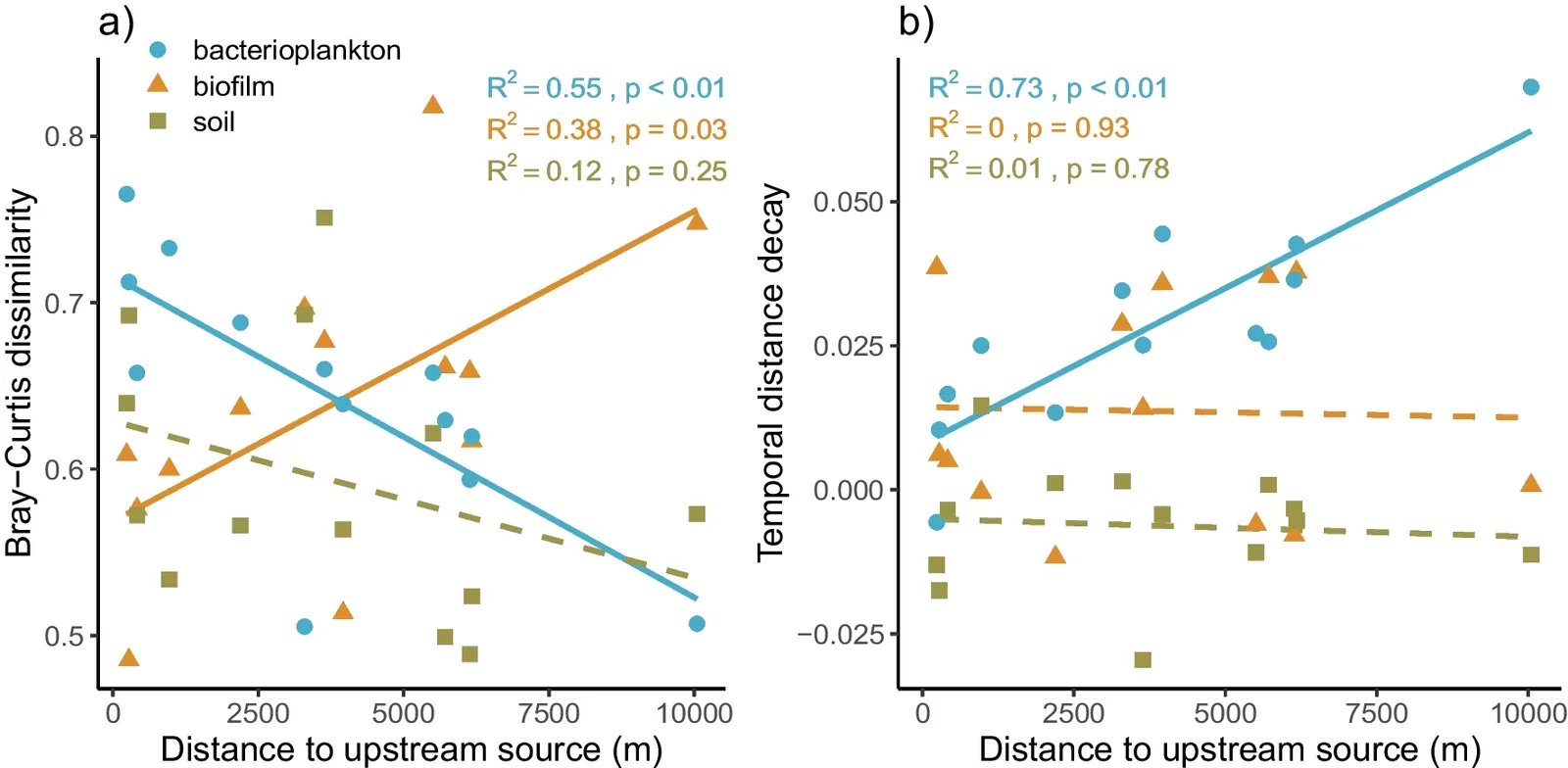
Temporal β-diversity (**a**) and temporal distance decay (**b**) for bacterioplankton, biofilm and riparian soil communities in relation to dendritic distance from the upstream source (i.e., position within the stream network). Site-specific values for temporal β-diversity were measured as mean Bray–Curtis dissimilarity across consecutive time points and those for the distance decay as a slope of linear regression between pairwise community dissimilarities and distance in time, respectively. Statistically significant linear regression patterns (*p* < 0.05) are displayed as solid lines and non-significant as dashed lines

The average temporal β-diversity was almost the same for biofilm (0.638) and bacterioplankton (0.644), whereas it was slightly lower for riparian soil communities (0.594). For biofilm bacteria, temporal β-diversity increased from headwaters towards the mainstem, i.e., headwater communities were more stable than downstream communities (Fig. 2a). For bacterioplankton, the pattern was opposite: temporal β-diversity peaked in headwaters and decreased towards downstream sites (Fig. 2a). However, pairwise dissimilarity in bacterioplankton composition was less dependent on the temporal distance between samples in headwater than in downstream sites where community dissimilarity clearly increased with distance in time (Fig. S3), creating a strongly positive relationship between temporal distance decay and network position (Fig. 2b). For biofilm communities, temporal distance decay was unrelated to network position (Fig. 2b, S3). Soil communities showed no response to stream network position either as temporal β-diversity (Fig. 2a) or temporal distance decay (Fig. 2b).

**Fig. 3:**
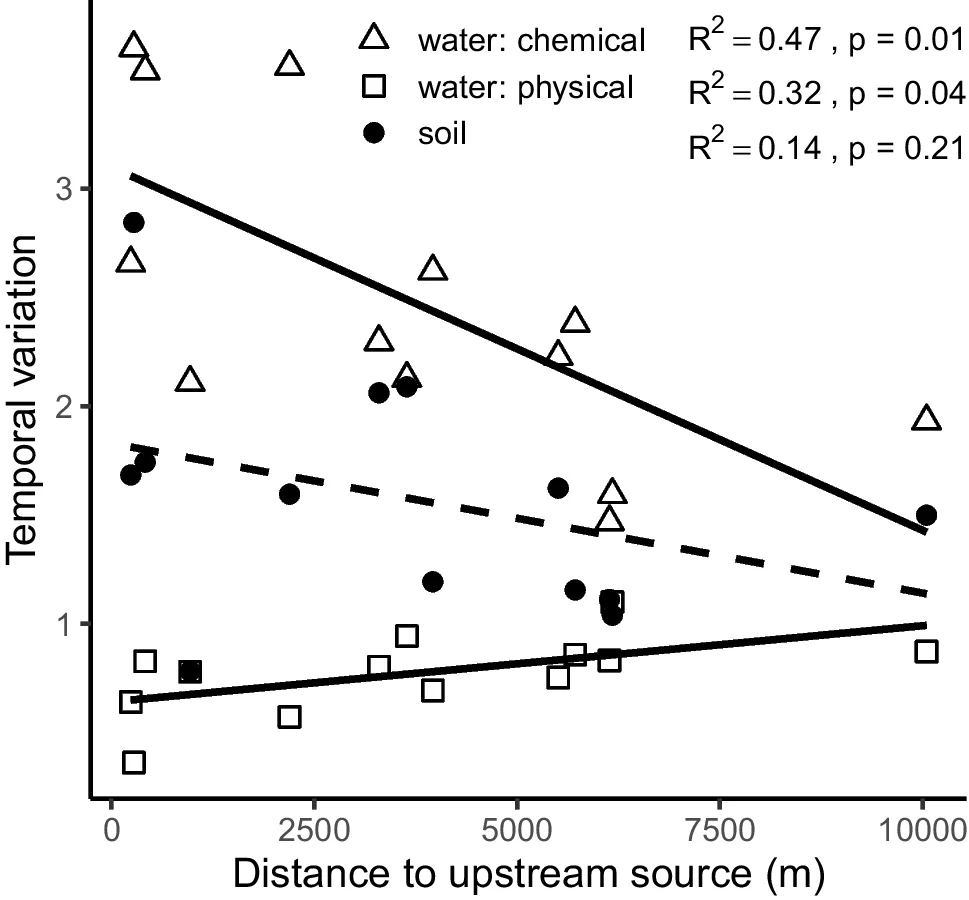
Temporal variation in the stream and soil environment in relation to distance from the upstream source (i.e., position within the stream network). Temporal variability was measured as site-specific mean Euclidian distance between consecutive sampling points based on water or soil chemistry and instream physical variables. Statistically significant linear regression patterns (*p* < 0.05) are displayed as solid lines and non-significant as dashed lines

**Fig. 4:**
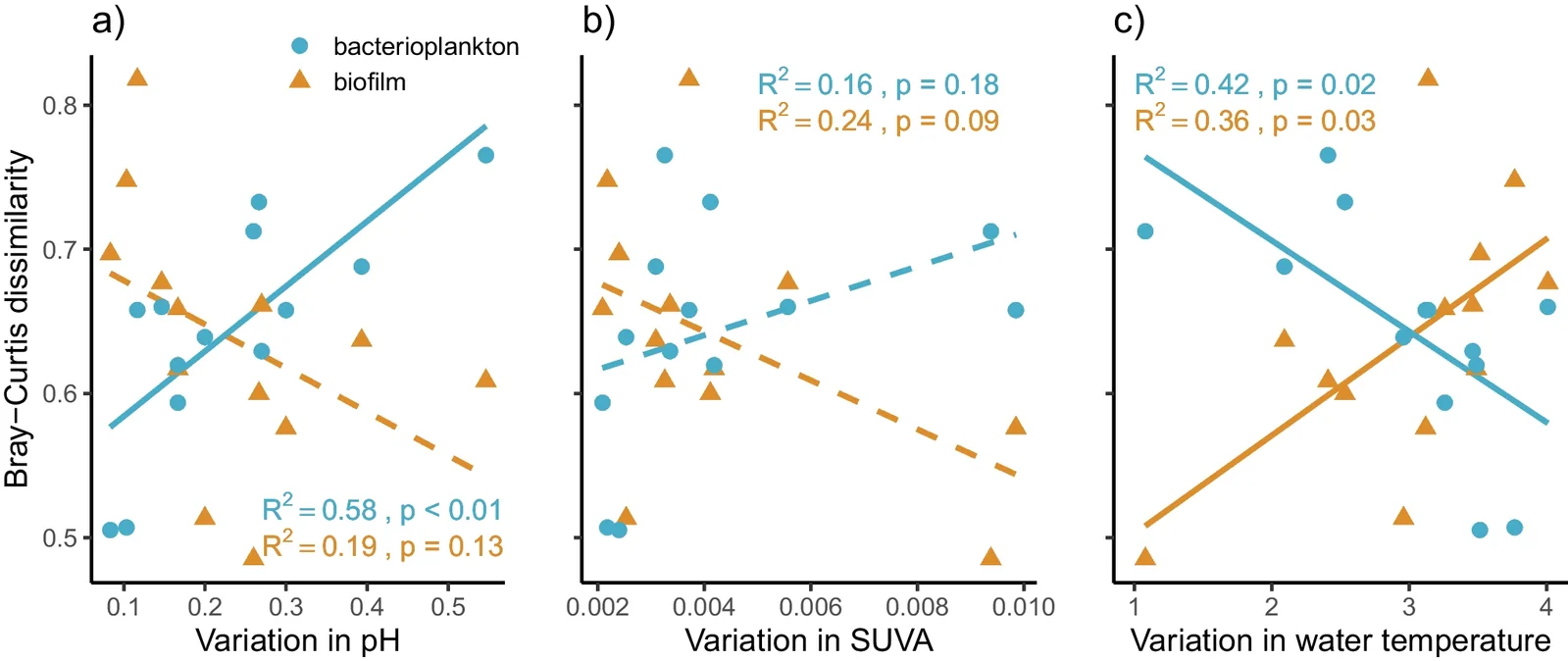
Site-specific temporal β-diversity (Bray–Curtis dissimilarity) for the two bacterial community types in relation to temporal variability in stream water pH (**a**), SUVA index (**b**), and water temperature (**c**). Temporal variability was measured as site-specific mean Euclidian distance between consecutive sampling points for each environmental variable separately. Statistically significant linear regression patterns (*p* < 0.05) are displayed as solid lines and non-significant as dashed lines

Based on Euclidian distance between consecutive sampling times headwater sites exhibited more temporal variation in water chemistry (pH, TotP, NO_2_ + NO_3_, and SUVA) than did the downstream sites, whereas physical factors (water temperature and level) varied slightly, but consistently more through time in lower river sections (Fig. 3). These spatial patterns were mainly driven by pH, SUVA, and temperature (Fig. S4); therefore, we focused on these variables as potential explanatory factors for biofilm and bacterioplankton community stability. Temporal β-diversity of biofilm communities and temporal variation in water pH or SUVA were weakly (non-significantly) negatively correlated (Fig. 4a, b), whereas there was a positive relationship between temporal β-diversity of biofilm and water temperature (Fig. 4c). For bacterioplankton, the patterns were opposite: positive relation with temporal variation in pH (Fig. 4a), and negative with variation in temperature (Fig. 4c). However, these patterns disappeared in partial regressions where the distance to stream source (i.e., network position) was kept constant (all *R*^2^ < 0.11, *p* > 0.267), except the near-significant trend between temporal variability in bacterioplankton and temporal variability in pH (*R*^2^ = 0.30, *p* = 0.053).

As expected, temporal variability of the soil environment (pH, conductivity, TotC, and N) was unrelated to river network location (Fig. 3). Temporal β-diversity of soil communities was strongly positively correlated with temporal variability of the soil environment (*R*^2^ = 0.69, *p* < 0.001). However, unlike for the aquatic community types, this pattern was not disproportionately controlled by any specific factor but N and C content and soil pH all contributed importantly (all *R*^2^ > 0.31, *p* < 0.05; Fig. S5).

**Fig. 5:**
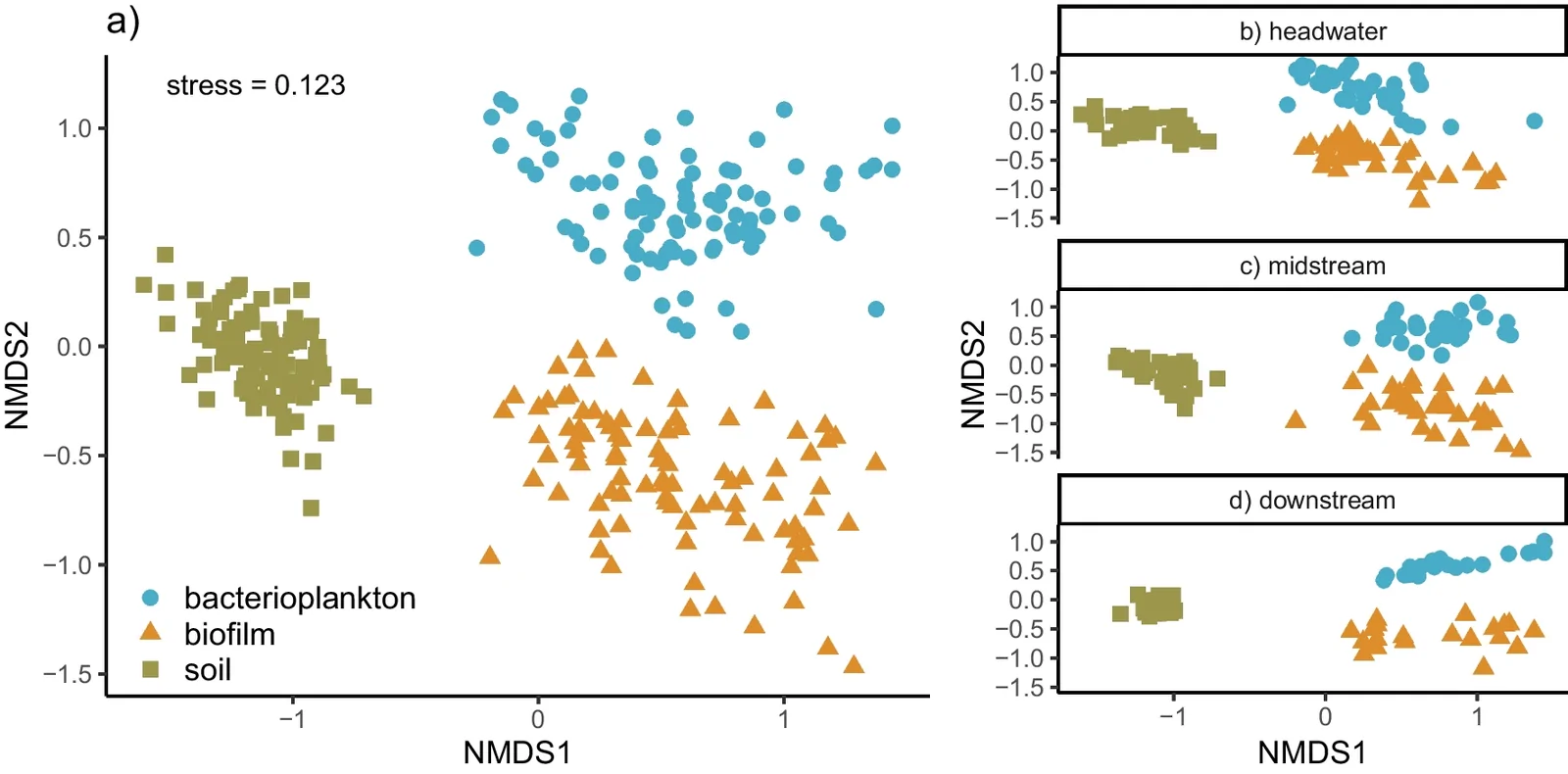
Non-metric multidimensional scaling (NMDS) of bacterial community composition based on Bray–Curtis dissimilarities across different community types for all samples (**a**), and the same ordination plotted separately for different stream network positions: headwaters (**b**), mid-stream (**c**) and downstream (**d**) sites

Water column and benthic biofilm supported distinctly different bacterial communities compared to riparian soil (biofilm vs. soil, PERMANOVA: *F*^1,180^ = 56.5, *p* = 0.001; bacterioplankton vs. soil: *F*_1,180_ = 58.3, *p* = 0.001; Fig. 5a). However, aquatic communities in headwaters were slightly more similar to soil communities than were those in downstream sections (Fig. 5b–d, Table S1). Compositional similarity to soil communities also varied more through time in headwater sites compared to lower stream reaches (Table S1).

**Fig. 6:**
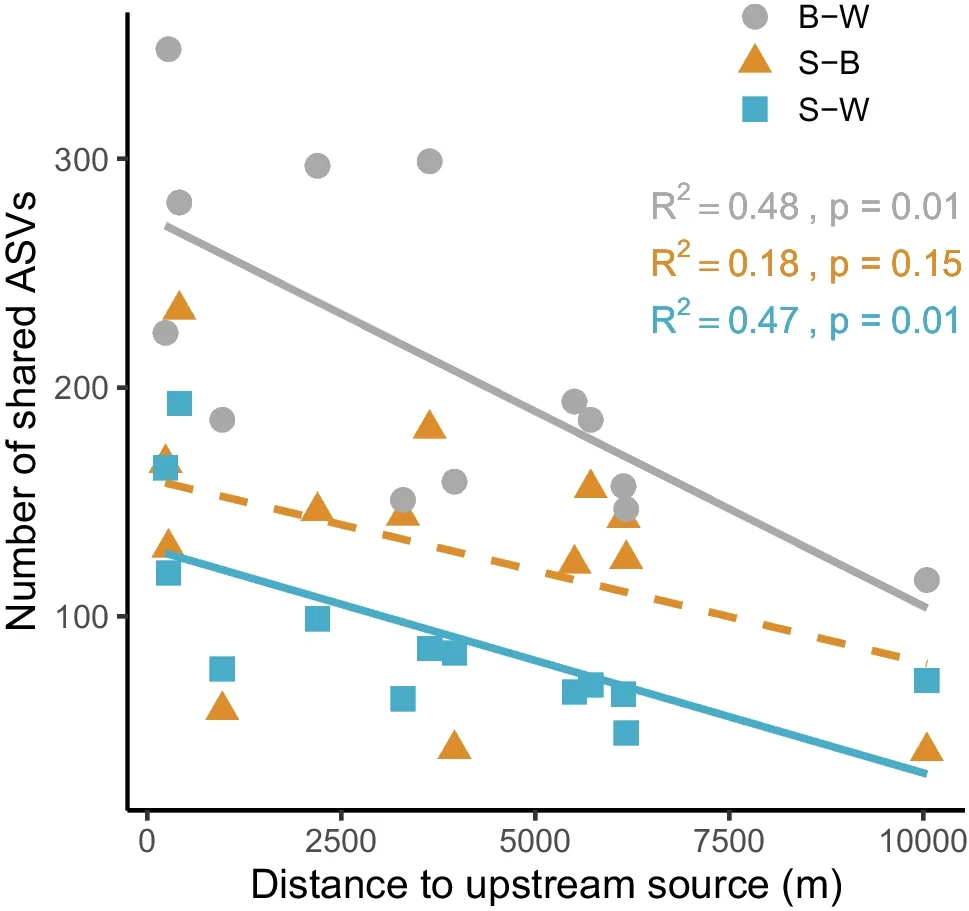
The mean number of ASVs shared between biofilm, bacterioplankton and riparian soil communities in relation to dendritic distance from the upstream source. B-W = biofilm and bacterioplankton, S-B = soil and biofilm, S-W = soil and bacterioplankton. Site-specific mean values were obtained by averaging across sampling times. Statistically significant linear regression patterns (*p* < 0.05) are displayed as solid lines and non-significant as dashed lines

The close linkage between the stream and riparian habitats in headwaters was also supported by a sharp reduction in the number of taxa shared between bacterioplankton and soil communities along the flow path (Fig. 6). A similar longitudinal pattern was detected for biofilm communities, but it was non-significant and strongly leveraged by the lowermost study site. Also the number of taxa shared between biofilm and bacterioplankton decreased from headwaters to downstream direction (Fig. 6). *Pseudomonadota* were the most abundant bacterial phylum among the ASVs shared between the soil and the aquatic habitats (Fig. S6d, e). *Verrucomicrobiota* and *Cyanobacteria* were rare in soil, but abundant among the shared soil-bacterioplankton and soil-biofilm ASVs, respectively. The most abundant phylum in soil, *Acidobacteriota*, were more abundant among the shared soil-biofilm than soil-bacterioplankton samples, and more abundant in headwaters than in mid- and downstream sections in both aquatic habitats (Fig. S6a, d, e). The shared ASVs generally occurred in very low abundances in soil but became relatively more abundant in the aquatic habitats, particularly in mid- and downstream sections, except one *Xanthobacteraceae* ASV which was more abundant in soil than in biofilm (Table S2). Interestingly, the number of ASVs shared with the soil was overall somewhat higher for biofilm than bacterioplankton communities (mean of 130 vs. 93 ASVs, corresponding to 11% vs. 9% of total biofilm or bacterioplankton ASVs, respectively).

## Discussion

Previous research on such widely differing organism groups as benthic invertebrates [8, 12] and bacterial biofilm [20] has shown that the upmost headwaters support more variable biological communities (higher spatial β-diversity) compared to downstream parts of the riverine network. Mechanisms generating this pattern are largely the same in each group: constrained dispersal caused by the dendritic nature of the stream network combined with higher environmental heterogeneity among headwater than downstream sites [9, 12]. Microorganisms are considered mainly passive dispersers [44], and therefore, their dispersal in stream networks is unidirectional, enhancing the potential role of dispersal limitation in generating higher β-diversity in the upmost headwaters [20]. Very few previous studies have examined whether headwater biotic communities also exhibit the highest temporal, in addition to spatial, β-diversity within the fluvial network. Our key finding was that bacterioplankton indeed had the highest temporal β-diversity at the upmost headwaters, whereas biofilm bacteria exhibited an overall weaker, but opposite pattern, with communities becoming temporally more variable along the flow path. In contrast to aquatic community types, riparian soil communities exhibited no spatial signal in their temporal variability, but were controlled by temporal variation in the soil environment.

The higher temporal variability of headwater bacterioplankton compared to mainstem sites appeared to result mainly from the variable input of propagules from the riparian soil, as the similarity to soil communities, although low overall, varied more through time in headwater than in downstream sites, and the number of taxa shared with soil communities decreased significantly across the network. These results emphasize the decisive role of the land–water connection and network position to bacterioplankton, but less so to biofilm community composition. The soil-derived “tourist” taxa that enter the stream at the upmost branches are gradually lost as the distance increases [17, 18, 21, 45]. These taxa apparently cross the land–water interface mainly during rainfall events [24, 46], and therefore, their imprint on local headwater communities is highly variable through time, resulting in high temporal β-diversity. As the linkage between the water and the riparian soil communities is transient [11], it may remain undetected in many sampling programs. In our study bacterioplankton, biofilm and riparian soil represented distinctly different communities across seasons and largely irrespective of the network position. This may partly reflect the relatively low stream flow during our study. Heavy rainfall events were infrequent and mostly happened soon after a sampling survey, and thus, almost 2 weeks before the next sample (Fig. S1). Nevertheless, the number of taxa shared with soil decreased sharply towards downstream sections, particularly for bacterioplankton, providing evidence for the gradual weakening of the stream-forest interface as the stream gets wider. Similarly, the number of taxa shared between bacterioplankton and biofilm decreased to downstream direction. Interestingly, and against our expectation, biofilm shared generally more taxa with soil than did bacterioplankton, but as the number of shared ASVs was overall low this had no effect on the compositional distinctiveness of the bacterial community types.

We expected biofilm communities to be temporally less variable than bacterioplankton, and we also expected their temporal variation to be less dependent on network position. Thus, temporal β-diversity of biofilm was not expected to show a decreasing longitudinal trend similar to bacterioplankton. These patterns were expected because biofilm should not reflect inputs from the riparian soil as much as does bacterioplankton [11, 21]. Indeed, despite the spatial proximity of these two aquatic community types, the among-site variability (spatial β-diversity) of biofilm communities is reportedly lower than that of the water-column communities [21, 47]. However, the range of temporal β-diversity of biofilm and bacterioplankton assemblages in our study was about the same. While it should not be surprising that bacterial communities in the strongly seasonal subarctic streams are temporally variable [e.g., 21] it was somewhat unexpected that biofilm communities exhibited higher temporal β-diversity with increasing distance from the stream source. This pattern seemed to reflect temporal variation in water temperature, which also showed higher variability towards larger river sections. Similarly, temporal variability in water chemistry, especially pH which was linked to temporal β-diversity of bacterioplankton, was strongly dependent on stream network position. Thus, the two aquatic community types were both controlled by temporal variability of the stream environment, albeit via different environmental forces. However, the effects of stream network position on temporal β-diversity of bacterial communities seemed inherently entangled with the temporal variability of the stream environment across the network. The strong interaction between environmental drivers of community composition with stream network position has previously been observed also for benthic invertebrates [48]. Temporal variability in the soil environment and temporal β-diversity of soil communities were also strongly linked but, as expected, neither of them had any spatial pattern in relation to network position.

Another measure of temporal β-diversity, distance decay through time, yielded a different spatial pattern whereby bacterioplankton assemblages became clearly more dissimilar as a function of time but only in mainstem sites. This pattern is likely explained by the very high temporal variability of bacterioplankton assemblages at headwater sites across the temporal extent of our study (from 2 weeks to several months), resulting in almost flat temporal distance decay curve. Conversely, a distinct community shift with increasing temporal distance was detected in downstream sections, likely reflecting seasonal changes in community composition as a result of seasonally varying environmental conditions. Thus, opposite to what was recently observed for macroorganisms [49], bacterioplankton assemblages exhibited a strong spatial signal in their temporal distance decay, dictated by the dendritic river landscape. In contrast, biofilm and soil communities exhibited no consistent patterns of temporal distance decay. The difference between results from different measures of temporal β-diversity was also observed by Dallas et al. [49] highlighting the importance of choosing the best possible measure in relation to the research questions asked. In addition, the use of conceptually differing methods in different studies complicates their comparison and therefore requires careful consideration [50, 51].

Change and decline of biodiversity are among the most imminent challenges for the humankind, yet the pace and magnitude of the change are greatly understudied in all but few organism groups. Baseline information is needed to gauge the magnitude of anthropogenic change on ecosystems, yet the lack of such data from little-impacted reference systems is blatantly clear for microbes, the “unseen majority” [52], despite their key role in most ecosystem functions [53]. The dendritic nature of the fluvial network controls spatial patterns in stream bacterial diversity and, as shown in this study, network position and environmental filters associated with it also largely determine temporal β-diversity of bacterial communities. Importantly, temporal variation of the bacterioplankton and biofilm communities were often different, sometimes even opposite. Such differences between these two, physically contiguous microbial community types underscore the temporally variable linkage between in-stream and riparian soil bacteria, as well as the role of the upmost headwaters as “critical reservoirs” (sensu [20]) of biodiversity and metacommunity dynamics of stream microbes.

## Supplementary Information

Below is the link to the electronic supplementary material.

Supplementary file1 (PDF 767 KB)

## Data Availability

Raw sequence reads of individual samples with their corresponding metadata are deposited in NCBI SRA under BioProject #PRJNA821862. The BioSample file was constructed using MIMARKS Survey version 5.0 package.
