# Supplementary material for: Temporal Beta Diversity of Bacteria in Streams: Network Position Matters But Differently for Bacterioplankton and Biofilm Communities
Source: Microb Ecol. 2025 Apr 12;88:26. doi: 10.1007/s00248-025-02522-3 (PMC11992004; doi:10.1007/s00248-025-02522-3)
Supplement: Supplementary file 1 [file 248_2025_2522_MOESM1_ESM.pdf]

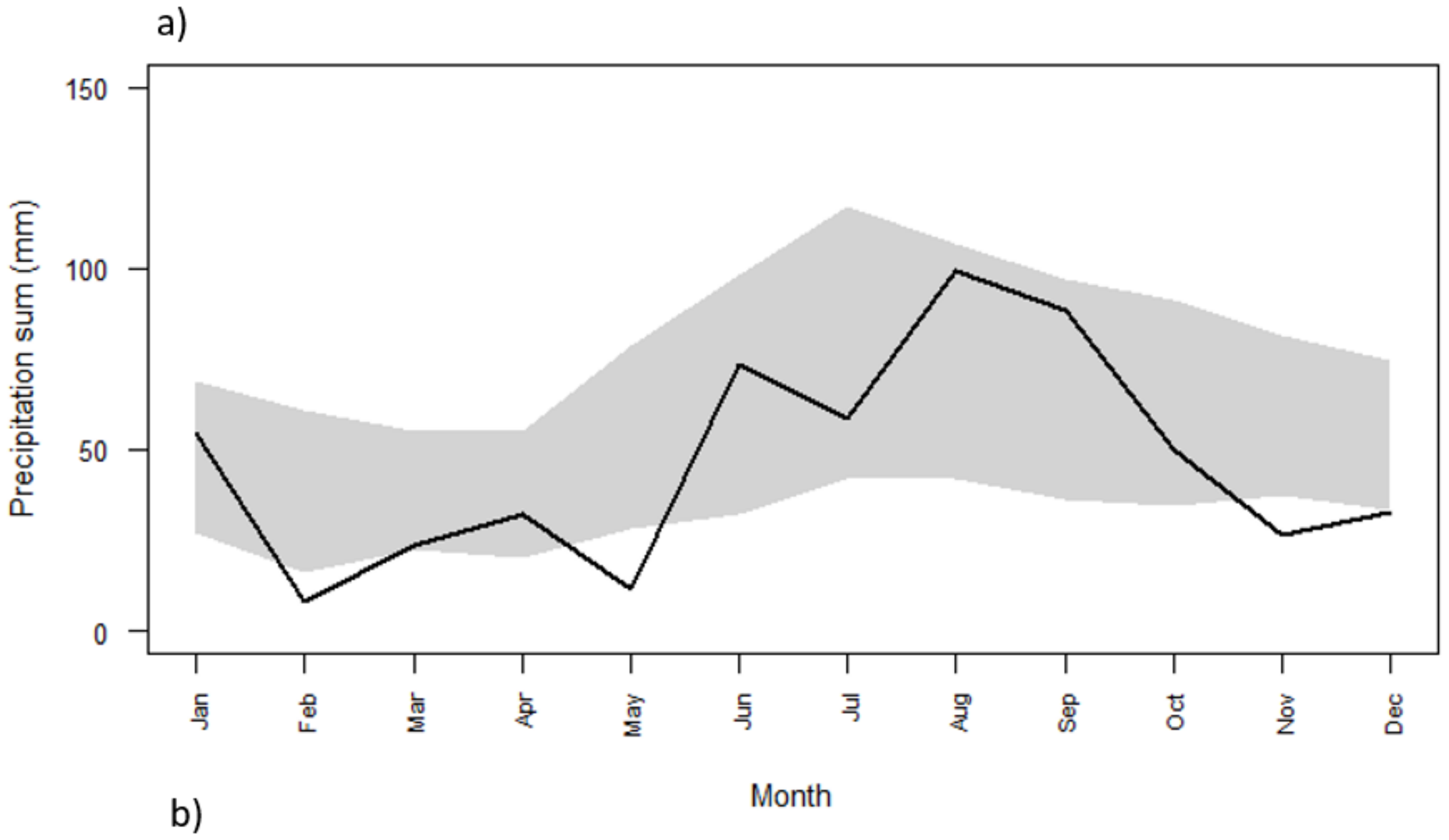

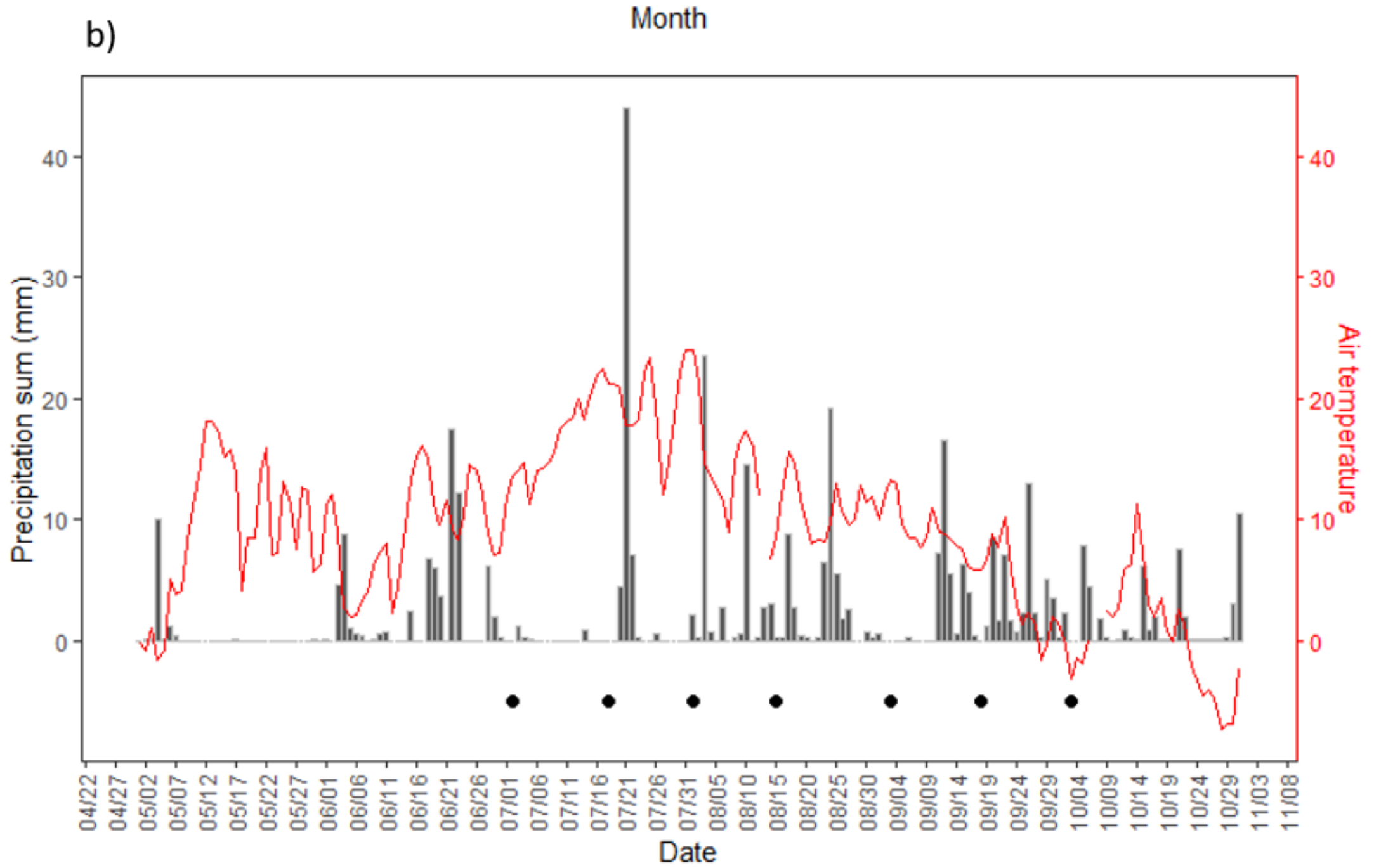

Fig. S1 a) Monthly precipitation sum (mm) in 2018 for the Riisijoki catchment area. Grey area shows the long term (since 1968) variability in precipitation calculated as monthly mean±1SD. b) Daily precipitation (mm) and air temperature (°C) from May to October. Sampling dates are indicated as black dots. Plots are based on climatic data obtained from the open database of Finnish Meteorological Institute (https://en.ilmatieteenlaitos.fi/download-observations).

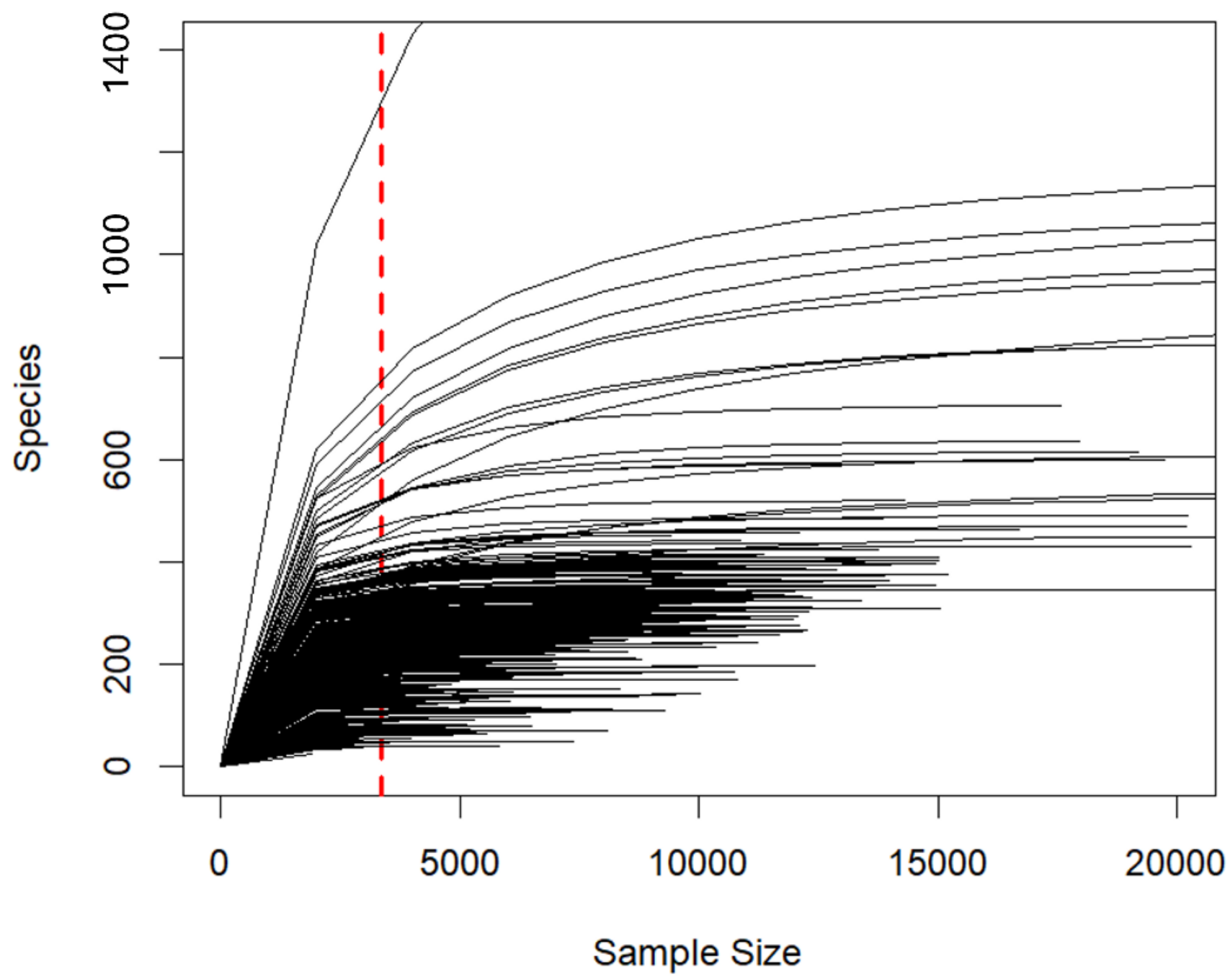

Fig. S2 Rarefaction curves for each sample. x-axis shows the increasing sequencing depth (´sample size') and y-axis the number of 'species', i.e. ASVs. Dashed red line shows the rarefied sequence depth used in the analyses.

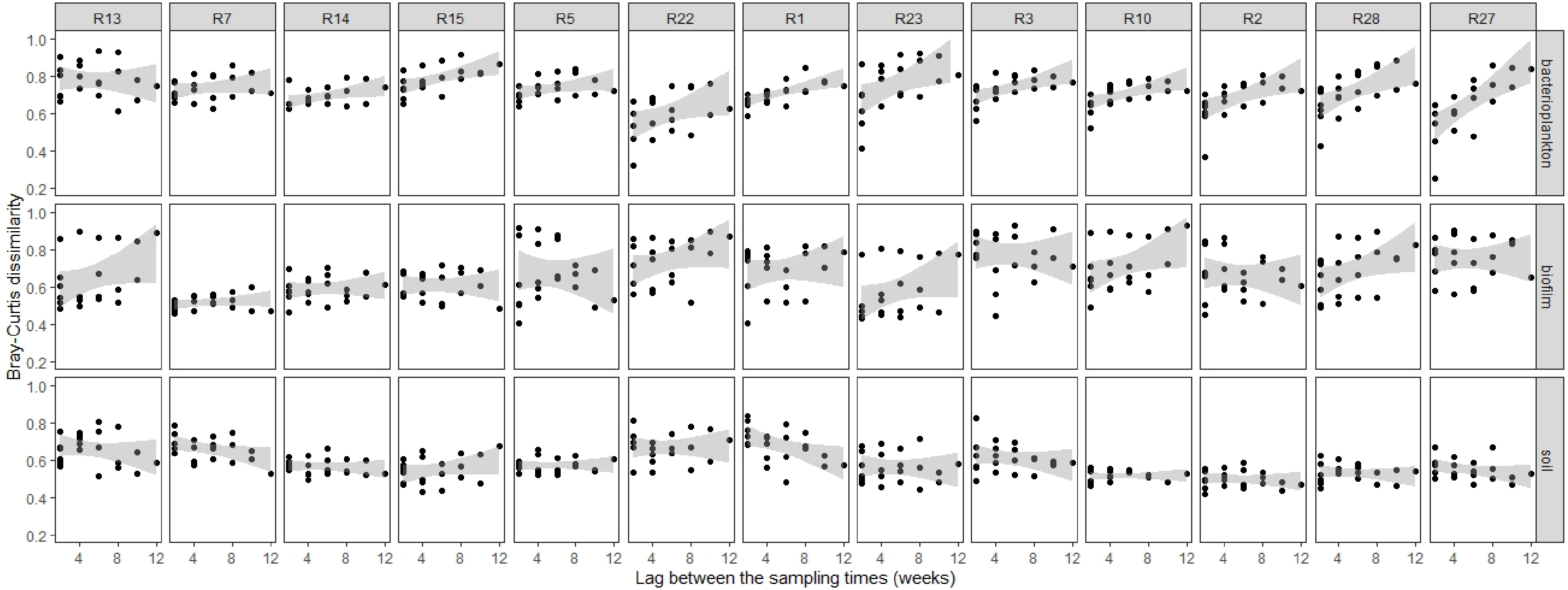

Fig. S3 Site-specific temporal distance decay of bacterial community similarity based on pairwise Bray-Curtis dissimilarity between sampling times for bacterioplankton, biofilm and riparian soil communities. Shaded areas represent confidence limits for linear regressions between dissimilarity and time lag between the samples. Temporal distance between the samples ranges from 2 to 12 weeks. Study sites are arranged in the order of increasing distance from the upstream source, i.e., headwater sites on left.

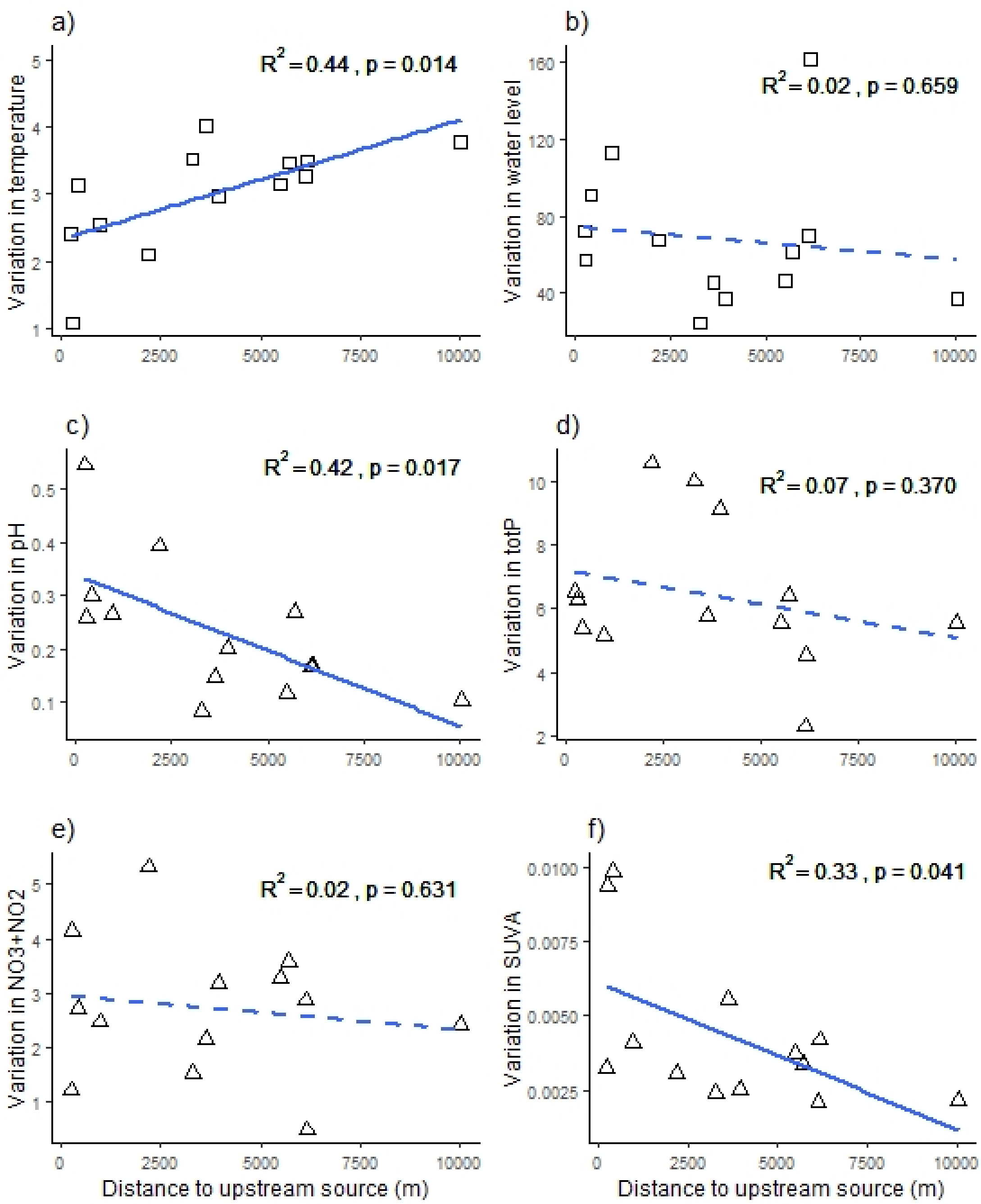

Fig. S4 Temporal variation in chemical and physical instream variables in relation to stream network position described by distance to the upstream source: water temperature °C (a), water level mm (b), pH (c), total phosphorus µg/l (d), nitrate-nitrite nitrogen µg/l (e), and specific ultraviolet absorbance SUVA (f). Temporal variation in each variable was measured as a site-specific mean of (unstandardized) Euclidian distances between the consecutive time points. Statistically significant linear regression patterns ($p < 0.05$) are displayed as solid lines and non-significant as dashed lines.

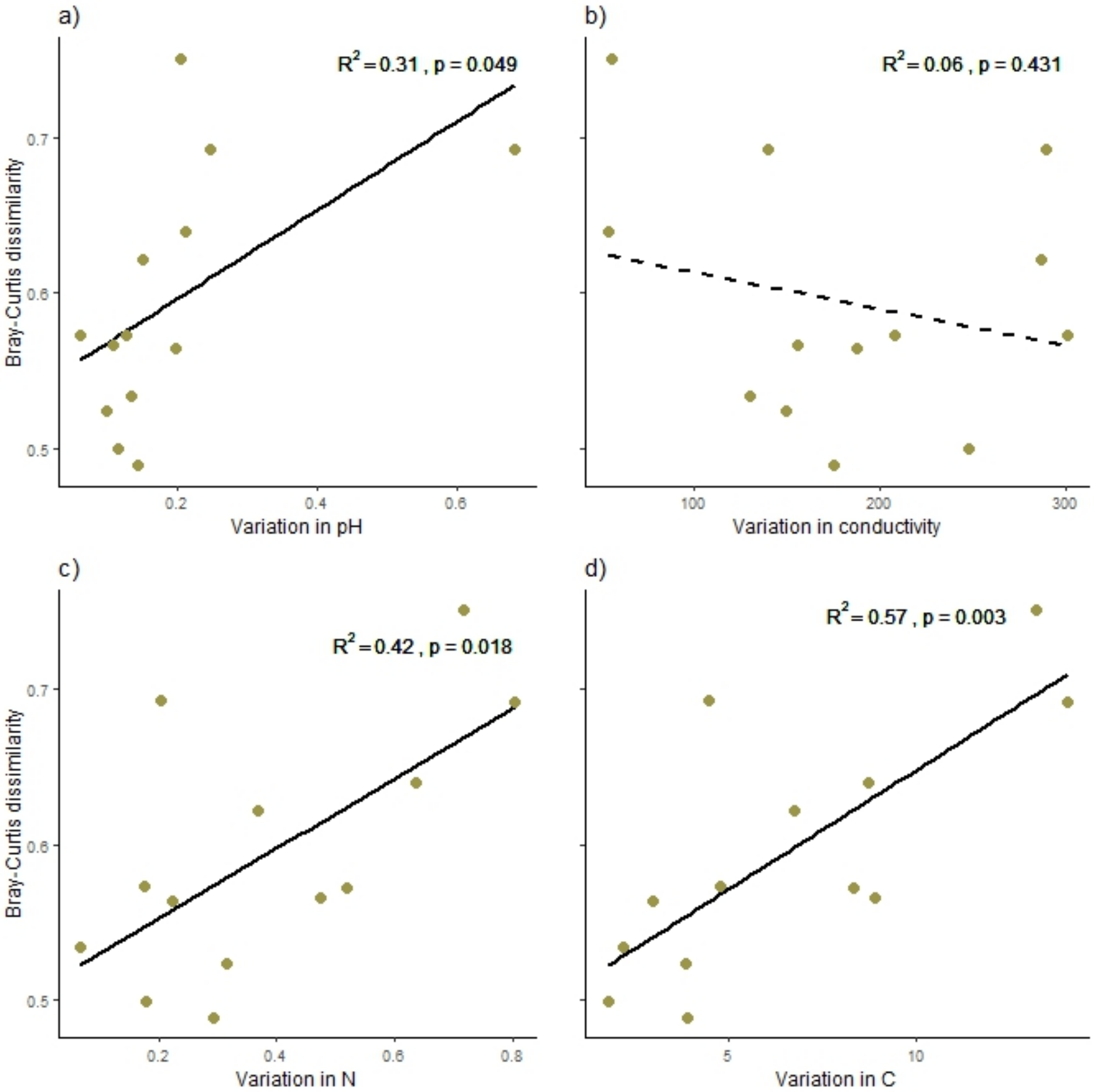

Fig. S5 Temporal β-diversity (Bray-Curtis dissimilarity) of soil bacteria communities in relation to temporal variability in soil pH (a), conductivity µS/cm (b), nitrogen (c) and carbon content %w/w (d). Temporal variability in the environmental variables was measured as site-specific mean (unstandardized) Euclidian distance between consecutive sampling points. Statistically significant linear regression patterns ($p < 0.05$) are displayed as solid lines and non-significant as dashed lines.

Table S1. Similarity of the aquatic community types to the surrounding soil communities, and the temporal variability in similarity, in different stream network locations. Similarity is expressed as a) average Bray-Curtis similarity and as b) distance between the group centroids in a 2-dimensional ordination (see Fig. 5). Temporal variability in similarity is expressed as a mean across site-specific standard deviations over sampling times. For each variable the values presenting highest similarity (a-b), or greatest variability (c) are bolded for each habitat type separately.

Community type	Position	a) Average similarity (Bray-Curtis)	b) Distance between the group centroids	c) Temporal variability in similarity (std)
Biofilm	Headwaters	**0.061**	**0.609**	**0.030**
	Midstream	0.037	0.635	0.020
	Downstream	0.040	0.692	0.022
Bacterioplankton	Headwaters	**0.056**	**0.597**	**0.025**
	Midstream	0.032	0.653	0.015
	Downstream	0.029	0.740	0.019

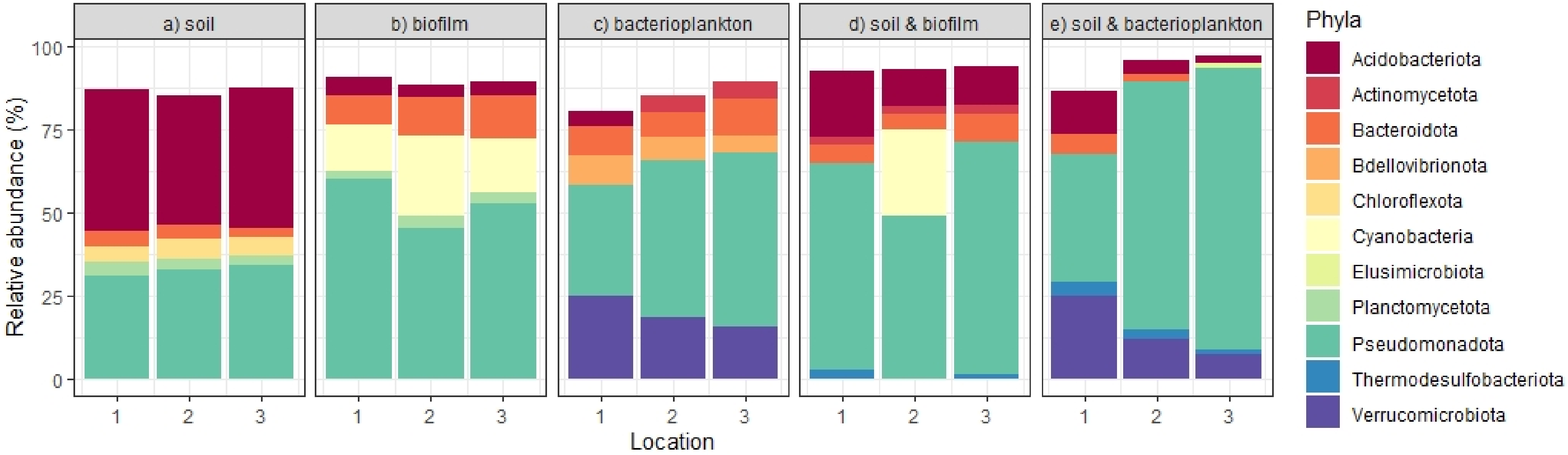

Fig. S6 a-c) Relative abundances of the five most dominant phyla in soil, biofilm, and bacterioplankton samples, respectively. The list of dominance was determined for each community type separately. d-e) Relative abundances of the shared ASVs between site-specific soil and biofilm (d) or soil and bacterioplankton (e) samples aggregated to the phyla level. Values in all panels (a-e) are averages across sites within each size class (location: 1=headwaters, 2 = midstream, 3=downstream).

Table S2. Mean abundance and occurrence of the most abundant shared, site-specific a) soil-biofilm ASVs and b) soil-bacterioplankton ASVs in relation to stream network position. Soil column presents the mean abundance and occurrence in soil samples. ASVs are in order of decreasing abundance based on upstream bacterioplankton / biofilm samples. The five most abundant ASVs are highlighted for each stream network position.

a)

	Relative abundance (mean across samples)						Occurrence (% of samples)					
	upstream		midstream		downstream		upstream		midstream		downstream	
Taxon (Phylum; Class; Order; **Family**; Genus; Species)	biofilm	soil	biofilm	soil	biofilm	soil	biofilm	soil	biofilm	soil	biofilm	soil
Pseudomonadota; Gammaproteobacteria; Burkholderiales; **Comamonadaceae**; Rhodoferax; NA	3.725	0.164	2.243	0.143	2.904	0.089	100	43	100	37	100	24
Pseudomonadota; Gammaproteobacteria; Burkholderiales; **Comamonadaceae**; Rhizobacter; NA	1.395	0.143	1.074	0.097	0.994	0.151	91	26	91	14	90	29
Bacteroidota; Bacteroidia; Cytophagales; **Spirosomaceae**; Arcicella; uncultured_bacterium	1.054	0.003	0.358	0.002	1.124	0.003	74	6	77	3	95	10
Acidobacteriota; Acidobacteriae; Acidobacteriales; **uncultured**; uncultured; uncultured_bacterium	0.993	0.338	0.523	0.486	0.344	0.401	94	69	83	86	71	95
Pseudomonadota; Alphaproteobacteria; Rhizobiales; **Xanthobacteraceae**; NA; NA	0.904	2.933	0.544	2.934	0.570	0.027	80	100	66	100	95	14
Cyanobacteria; Cyanobacteriia; Cyanobacteriales; **Nostocaceae**; Scytonema_UTEX_2349; NA	0	0	11.086	0.003	0	0	0	0	94	3	0	0
Pseudomonadota; Gammaproteobacteria; Burkholderiales; **Comamonadaceae**; NA; NA	0	0	1.399	0.014	0	0	0	0	83	3	0	0
Pseudomonadota; Gammaproteobacteria; Burkholderiales; **Burkholderiaceae**; Polynucleobacter; NA	0.142	0.018	0.390	0.038	0.570	0.027	37	9	86	23	95	14

b)

Taxon (Phylum; Class; Order; **Family**; Genus; Species)	**Relative abundance (mean across samples)**						**Occurrence (% of samples)**					
	**upstream**		**midstream**		**downstream**		**upstream**		**midstream**		**downstream**	
	**water**	**soil**	**water**	**soil**	**water**	**soil**	**water**	**soil**	**water**	**soil**	**water**	**soil**
Verrucomicrobiota; Omnitrophia; Omnitrophales; **Omnitrophales**; Omnitrophales; uncultured_planctomycete	3.362	0.053	1.903	0.118	1.990	0.121	91	23	71	34	67	43
Pseudomonadota; Gammaproteobacteria; Burkholderiales; **Comamonadaceae**; Rhodoferax; NA	2.847	0.164	2.434	0.143	2.132	0.089	86	43	100	37	100	24
Verrucomicrobiota; Omnitrophia; Omnitrophales; **Omnitrophaceae**; Candidatus_Omnitrophus; uncultured_planctomycete	1.738	0.046	1.295	0.018	1.025	0.011	89	14	71	11	67	5
Pseudomonadota; Gammaproteobacteria; Burkholderiales; **Comamonadaceae**; NA; NA	1.435	0.217	9.622	0.281	17.875	0.276	74	37	97	46	100	33
Verrucomicrobiota; Omnitrophia; Omnitrophales; **Omnitrophaceae**; Candidatus_Omnitrophus; uncultured_Banisveld	1.344	0.021	0	0	1.307	0.004	97	11	0	0	86	5
Pseudomonadota; Gammaproteobacteria; Burkholderiales; **Burkholderiaceae**; Polynucleobacter; NA	0.163	0.022	5.431	0.030	5.851	0.050	29	14	80	23	100	29
Pseudomonadota; Gammaproteobacteria; Burkholderiales; **Burkholderiaceae**; Polynucleobacter; NA	0.602	0.018	2.481	0.038	3.538	0.027	66	9	97	23	100	14
